# Drosha: a new tumor suppressor in pineoblastoma

**DOI:** 10.1101/gad.352932.125

**Published:** 2025-06-01

**Authors:** Zhixuan Huang, Xueli Ren, Jian Hu

**Affiliations:** Department of Cancer Biology, Cancer Neuroscience Program, The University of Texas MD Anderson Cancer Center, Houston, Texas 77054, USA

**Keywords:** *Dicer1*, *Drosha*, microRNA, pineoblastoma

## Abstract

To investigate the pathogenesis and target the vulnerability of human pineoblastoma, researchers have developed multiple genetically engineered mouse models that represent distinct molecular subtypes of the disease. In this issue of *Genes & Development*, Fraire and colleagues (doi:10.1101/gad.352485.124) examined the roles of key microRNA (miRNA) processing components Drosha and Dicer1. Loss of either *Drosha* or *Dicer1* partially mimicked the tumorigenic effects of *Rb1* deletion by promoting cell cycle progression through the derepression of Plagl2 and cyclin D2. This work reveals a novel mechanism of pineoblastoma development driven by disrupted miRNA processing and highlights a potential therapeutic strategy targeting downstream proliferative drivers.

## Abstract (precis)

In this Outlook, Huang et al. discuss a study in this issue of *Genes & Development* by Fraire et al. that shows that a deficiency in miRNA processors Drosha and Dicer and consequent cell cycle gene derepression promote pineoblastoma development. The authors highlight the heterogeneity of pineoblastoma's pathogenic mechanisms and its implications for therapeutic interventions in the clinic.

Pineoblastoma (PB) is a rare but highly aggressive pediatric brain tumor that arises primarily in the pineal gland—a small, pinecone-shaped endocrine organ located deep in the brain that regulates circadian rhythms through melatonin secretion. The prognosis for PB is generally poor due to its rapid progression, high recurrence rate, and low overall survival. Clinical data reveal a stark age-dependent prognosis: Children diagnosed at age 5 years old or younger have a 5 year survival rate of only 15%, compared with 57% in older children (Tate et al. 2012). Despite significant advances in cancer therapy, including immunotherapy, PB remains recalcitrant to current treatment strategies. Surgical resection is the primary therapy, but its efficacy is often limited by the tumor's deep-seated location. Adjuvant therapies, such as radiotherapy or high-dose chemotherapy, may improve control but often result in severe neurocognitive decline and systemic toxicity, particularly in younger patients.

PB is a heterogenous disease. Uncovering novel pathogenic mechanisms is crucial not only for deepening our understanding of its biological diversity but also for developing targeted therapies that address the distinct tumor cell populations. Based on DNA methylation profiling, PB can be classified into four molecular subtypes: (1) RB1-altered, primarily affecting infants; (2) MYC/FOXR2-activated, also seen in infants and characterized by *MYC* activation and *FOXR2* overexpression; (3) miRNA processing altered_1, found in children and associated with mutations in *DICER1*, *DROSHA*, or *DGCR8*; and (4) miRNA processing altered_2, which generally occurs in older children and is linked to a more favorable prognosis (Louis et al. 2021). Although some cases of PB are driven by germline mutations and loss of heterozygosity in single genes, others appear to arise sporadically through the accumulation of multiple genetic risk factors. This molecular and clinical heterogeneity poses a significant challenge for single-agent tumor therapies. Consequently, treatment strategies that combine multiple approaches to target the specific biological vulnerabilities of diverse tumor cell populations may offer improved outcomes.

Approximately 5%–15% of PB patients harbor germline mutations in *RB1*. The RB1 protein plays a vital role in regulating cell proliferation, maintaining normal neuronal differentiation, and preventing oncogenic transformation. The *RB1*-altered subtype primarily occurs in infants and young children; notably, ∼5% of these patients also present with bilateral retinoblastoma, a hallmark ocular tumor of early childhood. In mouse models, germline inactivation of *Rb1* leads to embryonic lethality midgestation due to defects in erythropoiesis and neurogenesis. Conditional *Rb1* deletion in interphotoreceptor retinol binding protein (IRBP)-expressing lineages induces neuroendocrine tumor formation (Vooijs et al. 2002). Tumorigenesis is significantly accelerated when *RB1* loss is combined with *TP53* deletion, underscoring the synergistic tumor-suppressive functions of these genes. This interaction was demonstrated using the *WAP-Cre:Rb*^*flox/flox*^:*Trp53*^*flox/flox*^ mouse model, which develops metastatic PB with full penetrance and short latency. Furthermore, Philip and colleagues (Chung et al. 2020) showed that a stabilizing *Trp53* mutation (*Trp53-R270H*) markedly enhances metastatic dissemination, closely mimicking the human disease phenotype.

Among PB patients under the age of 3 years old, ∼45% are classified within the PB MYC/FOXR2 subtype, which is associated with rapid disease progression and poor prognosis. This aggressive subtype is driven by recurrent focal gains of the *miR-17/92* cluster, which promotes proliferation, angiogenesis, and cell survival (Jiang et al. 2025). The *MYC* oncogene encodes a transcription factor that regulates cell growth, proliferation, and apoptosis, whereas *FOXR2* is involved in neural differentiation. Although germline mutations in *MYC* are rare, amplification and overexpression of both *MYC* and *FOXR2* have been strongly implicated in PB pathogenesis. To date, no genetically engineered mouse models (GEMMs) have been developed that faithfully recapitulate this molecular subtype, underscoring a critical gap in preclinical model development.

Germline mutations in *DICER1*, *DROSHA*, and *DGCR8*, key components of the miRNA processing machinery, have been identified in children and, more rarely, young adults with PB, though with only moderate penetrance. Somatic alterations, including loss of heterozygosity (LOH) at *DICER1*, homozygous deletion of *DROSHA*, and duplication of *PDE4DIP*, have also been reported in sporadic PB cases (Sabbaghian et al. 2012; Snuderl et al. 2018). Chung et al. (2020) previously reported that *Dicer1* ablation by WAP-Cre drove PB development in mice. Despite these findings, the mechanisms by which miRNA pathway dysregulation contributes to PB remain incompletely understood. Recent work by Fraire et al. (2025) demonstrated that conditional deletion of *Drosha* or *Dicer1* in *Trp53*-deficient, IRBP-expressing cells recapitulates features of *Rb1*-driven tumorigenesis. These tumors exhibit global miRNA loss—particularly within the *let7/miR-98-5p* family—and the resultant derepression of target genes, including those involved in S-phase progression and pineal-specific transcriptional regulation (Fraire et al. 2025). This study is the first to demonstrate the tumor-suppressive function of Drosha in a GEMM and uncovers a novel signaling axis promoting PB development: *let7/miR-98-5p* → *Plagl2* → *Ccnd2*. Consistently, previous research has shown that overexpression of D-type cyclins is sufficient to drive PB formation in a p53-null background (Idriss et al. 2023). The findings by Fraire et al. (2025) highlight a promising therapeutic strategy for targeting PB with Drosha/Dicer1 alterations by disrupting the proliferative signaling cascade.

PB remains a formidable clinical challenge, particularly in subtypes characterized by *RB1* loss and *MYC/FOXR*2 activation, which exhibit exceptionally poor clinical outcomes. Although recurrent mutations such as loss of *RB1*, *DICER1*, *DROSHA*, and *TP53* have been linked to distinct subtypes, numerous sporadic cases likely arise through yet undefined pathways. Although GEMMs have provided valuable insights and recapitulate certain aspects of the disease, they often fall short of capturing the full spectrum of tumor heterogeneity and the complexity of the tumor microenvironment. To address these limitations, it is essential to integrate complementary model systems, including human-derived cancer cell lines, tumor organoids, tissue slice cultures, and patient-derived xenografts, as critical platforms for cross-validation and to enhance translational relevance. Moreover, the development of more sophisticated, subtype-specific GEMMs, combined with advanced technologies and integrative, multidisciplinary approaches, will be key to deepening our understanding of the molecular mechanisms and pathogenesis of PB. Last, PB is notoriously resistant to standard treatments. The role of immune cells within the tumor microenvironment, particularly in mediating drug resistance and disease progression, remains unexplored and represents a promising avenue for future research.
